# Belief system, meaningfulness, and psychopathology associated with suicidality among Chinese college students: a cross-sectional survey

**DOI:** 10.1186/1471-2458-12-668

**Published:** 2012-08-17

**Authors:** Jiubo Zhao, Xueling Yang, Rong Xiao, Xiaoyuan Zhang, Diane Aguilera, Jingbo Zhao

**Affiliations:** 1 Department of Psychology, School of Public Health and Tropical Medicine, Southern Medical University, Guangzhou Dadao North Road 1838, Guangzhou, China; 2 School of foreign studies, Southern Medical University, Guangzhou Dadao North Road 1838, Guangzhou, China

**Keywords:** Suicidality, Religion, Political belief, Meaningfulness, Psychopathology, China

## Abstract

**Background:**

Research suggests that Chinese religious believers are more likely to commit suicide than those identifying as non-religious among rural young adults, contrary to findings in Western countries. However, one cannot conclude that religiosity is associated with elevated suicide risk without examining the effect of political and religious beliefs in a generally atheist country like China where political belief plays a dominant role in the belief system of young adults. The present study investigated the effects of political and religious belief on suicidality with meaningfulness and psychopathology as potential mediators in a large representative sample of Chinese college students.

**Methods:**

A cross-sectional survey was conducted among 1390 first-year college students randomly sampled from 10 colleges and universities in mainland China.

**Results:**

A total of 1168 respondents (84.0%) provided complete data on all variables. Lifetime prevalence of suicidal ideation, plan, and attempt were 45.1%, 6.8%, and 1.9% respectively, with one-year suicidal ideation showing at 19.3%. Female gender was associated with elevated risk of suicidality. Political belief but not religious belief was associated with decreased suicide risk. A significant interactive effect of political belief and religious belief was found, indicating that for political believers, being religious was associated with decreased suicide risk; for non-political believers, being religious was associated with increased suicide risk. Multi-group structural equation modeling showed that meaningfulness completely mediated and psychopathology partially mediated the effect of belief system on suicidality. Gender differences were found in pathways of political belief by religious beliefs to suicidality and political belief to psychopathology. The coefficients were significant for males but not for females.

**Conclusions:**

In less religious societies, political belief may serve as a means of integration as does religious affiliation in religious societies. Males were more likely to benefit from the protective effect of a belief system on suicidality than females.

## Background

Suicide is now the leading cause of death among Chinese young adults aged 15–34 years, accounting for 19% of all deaths in this age group annually [1]. Mental disorders, particularly depression, substance-related disorders, and disruptive behavior disorders are associated with 88.6% of completed suicide among young people worldwide [2]. However, only a small proportion of those with mental disorders actually die by suicide. Furthermore, only approximately half of Chinese suicide cases are diagnosed with any mental disorders [3,4], suggesting that other important factors are involved in the path from psychopathology to suicidality in Chinese young adults.

Key constructs should be defined before the introduction of major findings. Most experts agree that suicide is a continuum of suicidal ideation, planning, attempt and completion [5,6]. Therefore, suicidality we discuss includes suicidal ideation, planning, non-fatal suicide attempt, and completed suicide.

Previous findings suggest that suicide results from many complex socio-cultural and personal factors. One of these is religiosity. The relationship between religiosity and decreased suicide rates has been well documented in Western societies [7,8]. Recent studies have found that religious affiliation is associated with less suicide risk in depressed inpatients, suggesting that moral objections to suicide and lower aggression level in religiously affiliated subjects might function as protective factors against suicide attempts [9]. Another study found that religious attendance was associated with decreased level of suicide attempt and remained significant after adjusting for social support in a nationally representative sample in Canada [10]. It is not clear, however, to what extent individuals who identify themselves with a high degree of religious belief, per se, contribute to a decreased suicide rate.

China is an officially atheist country with one of the lowest rates (less than 10%) in the world of people who consider themselves religious [11,12]. Religious believers are still treated as a minority population because of their small number and political disadvantage. In a psychological autopsy study conducted in Chinese rural young adults, it was found that religiosity was associated with increased suicide intent, contrary to findings in Western cultures [13]. In an attempt to explain suicidality in Chinese rural areas, Zhang et al. [14] argued, in his psychological strain theory, that churchgoers in China might experience more value conflict within the mainstream Chinese culture, and such mental strain could lead to elevated suicide risk. Complementing Zhang et al.’s work, another study found that joining the Communist Party or Communist Youth League was a protective factor against suicide among Chinese rural young adults [15]. The interpersonal psychological theory of suicidality proposed by Joiner [16], which stresses the role of joint occurrence of perceived burdensomeness and failed belongingness in individuals who develop suicidal ideation, could also provide insight into the association of suicidal intent and religiosity. It illustrates why belonging to the Communist Party could serve as protection against suicide. However, the above studies were conducted mainly in Chinese rural populations. We cannot infer that religiosity is associated with elevated suicide risk in China without examining the interactive effect of political belief and religious belief in a diversified sample.

As is well known, China’s political system is characterized by a communist party administration, with communism and socialism being the mainstream ideologies nationwide since 1949 [17]. Contemporary Chinese youth, religious or non-religious, are growing up with the development of political belief as a main aspect of their self-identity. Historically, Confucianism, Buddhism, and Taoism have greatly shaped Chinese society [18]. Confucianism is not considered a dominant religion in this article because compared with Western religions, contemporary Confucianism is characterized by no religious rituals, is less institutionalized, and does not involve belief in an afterlife or Supreme Being. It is instead a way of life in connection to society [15]. Confucianism, with its moral, social, political, philosophical, and quasi-religious thought, has had a far-reaching influence on Chinese history and culture. In Confucianism, human beings are teachable and perfectible through personal and communal endeavors. Confucianism upholds the cardinal moral values of *ren* (humanity) and *yi* (righteousness) in achieving personal perfection with meaningful lives [19]. Modern China witnessed a decline in the popularity of Confucianism, but in the past 20 years there has been a renaissance of Confucianism in its political system [20]. Nowadays, Confucianism remains the main philosophy that syncretizes well with Marxism, shaping Chinese youths' moral and political beliefs [21]. The construction of a harmonious society emphasizing overall societal balance and harmony, promoted by the 2005 National People's Congress, has been the most important recent development in socialism with Chinese characteristics theory and is consistent with the Confucian cardinal notion of *tian ren he yi* (nature and man are an integral whole) [22].

Questions about what contemporary Chinese youths believe in and how to cultivate appropriate belief system among young people have been a continuous focus of Chinese educators’ and sociologists’ attention [23,24]. However, the relationship between belief system, especially political belief and mental health, remains unexplored. The main reason for this is that the concept of a belief system is so abstract that it is hard to measure and define precisely. To clarify terms in this study, operational definitions are provided as follows. Belief system is defined as a set of mutually supportive beliefs. The beliefs may be religious, ideological, philosophical, or a combination of these [25]. Political belief refers to a set of ideas held by a group of people that are used to understand the structure of forces in society, existing mechanisms of economic distribution, and conflicts in society (e.g., communism, socialism, and Confucianism in today’s China) [26]. Religious belief refers to a mental state in which faith is placed in a creed related to the supernatural, sacred, or divine. Such a state may be associated with the existence, characteristics, and worship of a deity or deities, divine intervention in the universe and human life, or values and practices centered on the teachings of a spiritual leader [18].

Another aim of the current study was to investigate other important factors that could shed light on the link between belief system and suicidality. One such factor is meaningfulness, a construct that relates to religiosity [27] and suicide [21,28] in the literature. Meaningfulness is defined as “perceived purpose and significance of life or existence in general” in this article. Previous research has found that meaningfulness is a protective factor against mental problems in Chinese young adults [29-31]. Another important factor is psychopathology, the most well-known risk factor for suicide [32,33]. Psychopathology is inversely related to meaningfulness in Chinese college students [34]. Illuminating the role of meaningfulness and psychopathology in pathways to suicidality could help to prevent suicide in young adults.

The principal purpose of the current study was to investigate the effect of belief system, including political and religious beliefs, as well as their interaction on suicidality in a nationally representative sample of Chinese young adults. The current sample focused on first-year college students, given that religious and political identities presumably become mature in late adolescence [35,36]. We aimed to test and modify structural equation models (SEM) to help in understanding the pathway to suicidality among variables of political belief, religious belief, meaningfulness, and psychopathology. The hypotheses of the current model were the following.1) Political belief and religious belief are directly associated with suicidality, although mediated by meaningfulness and psychopathology. 2) Psychopathology mediates the effect of meaningfulness on suicidality. 3) The interaction of political belief and religious belief impacts suicidality, although mediated by meaningfulness and psychopathology. 4) Female gender is associated with elevated suicide risk, as has been found in other Chinese studies [13,14,37]. Therefore, we assume that gender-related differences would have complex influences on suicidality that could be assessed by multi-group analyses using gender as the grouping variable.

## Methods

### Participants

Multistage cluster sampling was conducted to recruit 1390 first-year college students in mainland China. Five colleges and five universities were randomly sampled out of 1949 colleges and universities nationwide. The sampled schools included one out of 190 Project 211 universities (Project 211 is the Chinese government's new endeavor aimed at strengthening about 100 institutions of higher education and key disciplinary areas as a national priority for the 21st century), one out of 389 normal universities (those that train teachers, chiefly for the elementary grades), one out of 91 medical universities, one out of 160 business and finance colleges, one out of 130 colleges of science and technology, one out of 81 art colleges, one out of 299 universities administered by the Ministry of Education, one out of 118 provincial colleges, one out of 169 universities of agriculture and forestry, and one out of 403 other colleges. Then, a cluster sampling procedure was conducted to select classes in each of the 10 colleges and universities. Once selected, we sought to recruit all individuals within the cluster that had been chosen. Specifically, 24 classes were selected randomly from the potential pool of all classes. From the 24 classes, 1390 first-year students aged 16–24 years were identified as possible subjects.

Pooling all classes, the response rate was 84.7% (1177 out of 1390). Reasons for non-response included non-attendance of the survey class (207 students) and withdrawing before the questionnaire was completed (6 students). Cases marked by missing data on any of the variables under study were deleted from the analysis. Complete data on all variables were available from 1168 respondents (84.0%).

### Procedure

Data were collected from September to December 2010. Approval was obtained from the Ethical Committee of Southern Medical University. Before the formal study, a pilot study was conducted in 400 first-year medical students to help formulate study hypotheses and modify questionnaires. We took the following steps to control the quality of the survey and to address the ethical issues in the formal study: 1) improved the survey instrument through pilot study (e.g., based on the pilot survey, we re-arranged sensitive items regarding suicidality in the front to middle part of the survey); 2) standardized instruction and trained ten surveyors, one in each college; 3) carefully selected appropriate survey time to avoid the influence of unexpected stressful life events such as examinations; 4) cooperated with the administrative team of the sampled colleges to ensure that the survey environment was quiet and the participants sat at one-seat intervals; 5) assured the survey was kept voluntary, anonymous, independent, and confidential; 6) asked students for verbal consent to participate and gave them the option to withdraw at any time; 7) clarified ambiguous items before distribution of the questionnaire (e.g., political beliefs); 8) asked participants to re-check for missing responses before collection of questionnaires; 9) encouraged participants to list at least one of three means of contact, including email, phone number and qq number (a popular instant messaging tool in China) so feedback on their mental health profile could be provided. Once serious suicide risk was detected (e.g., recent detailed suicidal plan) we would contact him or her to provide personalized treatment advice and help-seeking resources.

### Measures

The survey instrument was a five -page questionnaire requiring about 30 minutes to complete. It included:

***Socio-demographic characteristics of the participants*** included gender, age, city, ethnicity (Han nationality coded 1, other ethnic minority coded 0), household registration (rural area = 1, urban area = 0), perceived family finance (good = 1, mediocre = 2, bad = 3), father’s occupation, mother’s occupation, marital status, whether from single-parent family, whether an only child, and number of siblings if any.

***Political belief*** was a dichotomous variable derived from two rounds of qualitative interviews and previous literature [22,38]. First, 50 third-year college students were interviewed to obtain responses to the open-ended question, “What do you believe in politically?” Responses were coded by two independent research associates. Results showed that the responses varied, including socialism with Chinese characteristics (34%), Confucianism (26%), no belief (18%), individualism (10%), capitalism (4%), skepticism (4%), and others (4%). For example, one student responded: “I don’t believe in any specific political propaganda; however, I have my own personal core values, such as happiness.” That response was classified as individualism. Second, 40 college students were asked during regular class time to reflect on the question, “What are the most important elements that affect you politically and culturally?” The responses were again coded by two independent research associates based on the above classifications (i.e., socialism, communism, Confucianism, individualism, capitalism, and skepticism) and sorted by frequency. Results showed that the most frequent responses were socialism, communism and Confucianism.

On the basis of the two rounds of interviews and previous literature, the final survey item was determined as “Do you think you have an explicit political belief of socialism with Chinese characteristics?”, No = 0, Yes = 1. (Note: Socialism with Chinese characteristics is an integration of basic Marxism principles, Confucianism-based Chinese traditional culture, and contemporary Chinese conditions. It contains Deng Xiaoping Theory^a^, the Important Thought of Three Represents^b^ and the Scientific Development Concept^c^ and other major strategic thoughts.) A total of 321 (27.5%) respondents replied "Yes" on this item.

***Religious belief*** was a dichotomous variable with respondents reporting beliefs in any of the dominant religions (Christianity, Buddhism, Islamism, Taoism, or Catholicism) coded 1 and no religion coded 0. A total of 126 (10.8%) respondents were religious believers: Christianity, 20 (1.7%); Buddhism, 70 (6.0%); Islamism, 5 (0.4%); Taoism, 28 (2.4%); and Catholicism, 3 (0.3%).

***Meaningfulness*** was assessed by the Purpose in Life scale (PIL), Chinese version [39]. The PIL consistsed of 20 statements rated on a seven-point scale with a high score (6–7) indicative of clear meaning and purpose, an intermediate score (3–5) representing indecision, and a low score (1–2) reflecting lack of clear meaning and purpose in life. A total score is calculated by summing the 20 ratings (total score ranges from 20 to 140). It has been validated in various samples [40]. Cronbach’s alpha was 0.89 for the current sample.

***Psychopathology*** was assessed by the Chinese version of the Symptom Checklist-90-Revised (SCL-90-R). The SCL-90-R measures participants’ self-reported psychopathologic features on nine subscales including somatization, obsessive-compulsiveness, interpersonal sensitivity, depression, anxiety, hostility, phobia, paranoid ideation, and psychoticism [41]. Each question is rated on a five-point Likert scale (1 for no distress, 5 for extreme distress). This instrument has been used extensively in studies measuring a variety of mental disorders. The reliability and validity of the Chinese version of the SCL-90-R has been established in previous studies [42]. In the current study, Cronbach’s alpha was 0.96.

***Suicidality*** was measured by the revised Suicidal Behaviors Questionnaire, Chinese version (SBQ-R) [43]. The SBQ-R has four items, each tapping a different dimension of suicidality: lifetime suicidal ideation (plan and attempt), prevalence of suicidal ideation over the past 12 months, threat of suicide attempt, and likelihood of suicide in the future. Specific items answered on a Likert-type scale are “Have you ever thought about or attempted to kill yourself?” (1–4), “How often have you thought about killing yourself in the past year?” (1–5), “Have you ever told someone that you were going to commit suicide, or that you might do it?” (1–3), and “How likely is it that you will attempt suicide someday?” (0–6). The total SBQ-R score ranges from 3 to 18, representing relative risks for suicidality on a continuum. Satisfactory psychometric properties have been reported in previous studies [44-46]. Cronbach’s alpha was 0.68 in the current sample.

### Statistical analyses

Statistical analyses were performed using SPSS 15.0 for Windows and AMOS 7.0 (SPSS Inc., Chicago, IL). First, a *t* test or *χ*^*2*^ test was used to identify statistically significant demographic differences associated with suicidality. Pearson product moment correlation was used to explore univariate associations between suicidality and independent variables including demographics, political belief, religious belief, meaningfulness, and psychopathology. Univariate analysis of variance was performed to test the interactive effect of political belief and religious belief on suicidality.

Multi-group analysis, a special case of SEM, was used to test the effects of gender differences on hypothesized models. Suicidality as a latent variable were assessed by lifetime suicidal ideation, plan and attempt, suicidal ideation over the past 12 months, suicide threat, and suicide possibility. Manifest variables included political belief, religious belief, political belief by religious belief and meaningfulness. Psychopathology was a latent variable with 10 indicators. Maximum likelihood estimation was employed as a global test of models. Model fit and comparison were ascertained using the following indices: root mean square error of approximation (RMSEA), comparative fit index (CFI), normed fit index (NFI), incremental fit index (IFI), Akaike information criterion (AIC), and expected cross-validation index (ECVI). Cutoff criteria for fit indices were determined following the recommendation of Hu and Bentler [47].

The tests of gender differences in the SEM framework start with estimating the hypothesized structure without constraining any parameter in both groups simultaneously (baseline model). An observed adequate fit of this model is required for further testing. All subsequent tests involve comparing a constrained model in which required parameters (i.e., factor loading, correlation paths, measurement residuals, and structural residuals) are equal for both groups with an unconstrained model that does not include the equality requirement. If the statistical fit of the constrained model reveals a significantly worse solution than the unconstrained one, this is interpreted as evidence for non-invariance. The fit of nested models can be compared by inspecting the significance of the change in *χ*^2^ values. In other words, when we force certain parameters to be equal, the significantly decreased model fit indices suggests that at least one of the parameters is different across groups.

## Results

### Characteristics of the study participants

The age of the sample was 19.47 ± 0.96 years (range 16–24). Among the 1168 participants who provided complete data, 542 (46.4%) were male, 626 were female (53.6%), 1127 (96.5%) were Han nationality, 372 (31.8%) were the only child of the family, 418 (35.8%) were from rural areas, and 750 (64.2%) were from cities or towns. As for perceived family financial status, 274 (23.4%) responded “Good”, 694 (59.4%) responded “Mediocre”, and 200 (17.1%) responded “Bad”.

### Prevalence of suicidality

The lifetime prevalences of suicidal ideation, plan, and attempt were 45.1%, 6.8%, and 1.9%, respectively. The prevalence of suicidal ideation over the last 12 months was 19.3%. Significant gender differences were found in SBQ-R total score (4.47 ± 1.86 for males, 4.97 ± 2.15 for females, *t* (1167) = −4.26, *P* < 0.001), lifetime prevalence of ideation (41% for males, 48.7% for females, *χ*^*2*^ = 7.07, *P* < 0.01), and plan (5.2% for males, 8.3% for females, *χ*^*2*^ = 4.49, *P* < 0.05). No significant gender difference was found in suicide attempt (1.8% for males, 1.9% for females, *χ*^*2*^ = 0.00, *P >* 0.05).

### **Univariate correlation among study variables**

The matrix of Pearson correlation coefficients is presented in Table 1. No demographic variables except gender were significantly related to suicidality. All independent variables except religious belief were significantly related to suicidality. In particular, meaningfulness and SCL-90 total score had the largest zero order correlation. Meaningfulness and suicidality had the second largest zero order correlation; that is, the greater the meaningfulness, the lower the suicide risks. Moreover,mean, SD, and inter-correlations among SCL-90 subscales can be can be found in Additional file 1.

**Table 1:** Inter-correlations among study variables

**Variables**	**Total sample (n = 1168, Mean ± SD)**	**Male (n = 542, Mean ± SD)**	**Female (n = 626, Mean ± SD)**	**1**	**2**	**3**	**4**	**5**
1 Gender	—	—	—	—				
2 Political belief	—	—	—	0.02	—			
3 Religion belief	—	—	—	−0.03	0.04	—		
4 SCL-90 Total Score	48.73 ± 34.95	49.85 ± 33.93	47.77 ± 35.81	0.03	−0.08^**^	0.02	—	
5Meaningfulness	100.33 ± 14.89	100.62 ± 14.50	100.08 ± 15.22	0.02	0.23^**^	0.01	−0.52^**^	—
6 SBQ-R Total Score	5.74 ± 2.04	5.47 ± 1.86	5.97 ± 2.15	−0.12^**^	−0.13^**^	0.03	0.38^**^	−0.44^**^

Note: ^*^p < 0.05, ^**^p < 0.01.

### Interaction between political belief and religious belief

**Figure 1:**
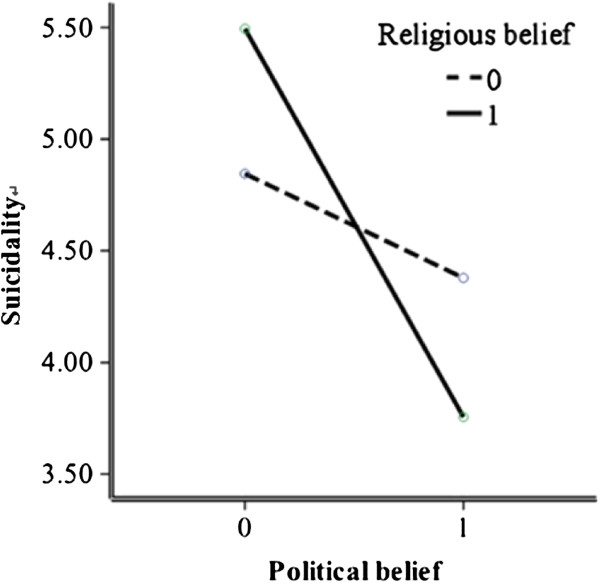
The interactive effect of political belief and religious belief on suicidality.

To test our hypothesis that political belief and religious belief had an interactive effect on suicidality, a univariate analysis of variance was performed with suicidality as the dependent variable, and political belief and religious belief as independent variables (fixed factors). The interaction term (political belief by religious belief) was added to the SEM model. As shown in Figure 1, there was a significant interactive effect of political belief by religious belief on suicidality, *F* (1,3) = 10.95, *P* < 0.001, *R*^2^ = .03, indicating that for political believers, being religious was associated with decreased suicide risk; for non-political believers, being religious was associated with increased suicide risk.

### Gender differences and the role of meaningfulness and psychopathology: multi-group structural equation modeling

The overall fit indices of the unconstrained model (baseline model) indicated that the observed data from both gender groups fitted the hypothesized model (see Table 2), *χ*^2^ = 614.16, *df* = 240, *χ*^*2*^/*df* = 2.56, RMSEA = 0.04, CFI = 0.96, NFI = 0.94, IFI = 0.96. Finally, AIC and ECVI, used to facilitate model comparisons, were 890.16 and 0.76, respectively.

**Table 2:** Goodness of fit indices for model comparisons

**Model**	***χ***^**2**^	***df***	***χ***^**2**^***/df***	**Δ*χ***^**2**^	**Δ*df***	***P value for *Δ*χ***^**2**^	***RMSEA***	***CFI***	***NFI***	***IFI***	***AIC***	***ECVI***
Threshold for acceptable fit			<5			≥0.05 (Significant Level)	<0.05	≥0.90	≥0.90	≥0.90		
Unconstrained	614.16	240	2.56				0.04	0.96	0.94	0.96	890.16	0.76
Measurement weights	655.61	252	2.60	41.44	12	0.00	0.04	0.96	0.94	0.96	907.61	0.78
Structural weights	669.54	264	2.54	55.37	24	0.00	0.04	0.96	0.94	0.96	897.54	0.77
Released correlation paths model	665.74	261	2.55	51.57	21	0.00	0.04	0.96	0.94	0.96	899.74	0.77

Note: *χ*^2^ = chi-square; *df* = degree of freedom;*χ*^2^*/df* = The chi-squared/freedom ratio;RMSEA = Root mean square error of approximation; CFI = Comparative fit index; NFI = Normed fit index; IFI = Incremental fit index; AIC = Akaike information criteria;ECVI = Expected cross-validation index.

The chi-square deference (Δ*χ*^2^) tests are compared with the unconstrained model (baseline model).

The next multi-group model constraining factor loadings (measurement weights invariance model) also yielded adequate data-model fit (see Table 2), *χ*^2^ = 655.61, *df* = 252, *χ*^2^/*df* = 2.60, RMSEA = 0.04, CFI = 0.96, NFI = 0.94, and IFI = 0.96. However, the data-model fit significantly changed compared with that of the unconstrained model, Δ*χ*^2^ = 41.44, Δ *df* = 12, *P* = 0.000, AIC = 907.61, ECVI = 0.78.

The structural weights invariance model constrained all paths between males and females to equal. The fit of this model was adequate, *χ*^2^ = 669.54, *df* = 264, *χ*^2^/df = 2.54, RMSEA = 0.04, CFI = 0.96, NFI = 0.94, IFI = 0.96. However, the data-model fit significantly changed compared with that of the unconstrained model, Δ*χ*^2^ = 55.37, Δ *df* = 24*, P* = 0.000, AIC = 897.54, ECVI = 0.77.

**Figure 2:**
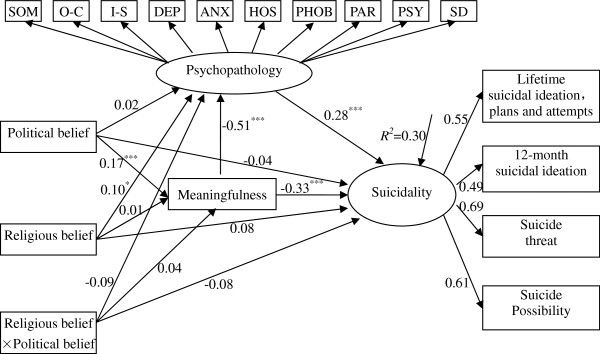
**Standardized structural coefficients for belief system, meaningfulness, psychopathology associate with suicidality among male Chinese college students.** Note: ^*^p < 0.05, ^**^p < 0.01, ^***^p < 0.001. *χ*^2^ = 614.16, *df =* 240, *χ*^2^*/df =* 2.56, *RMSEA =* 0.04, *CFI =* 0.96, *NFI = 0*.94, *IFI =* 0.96. SOM – Somatization; O-C - Obsessive-Compulsive; I-S - Interpersonal Sensitivity; DEP – Depression; ANX – Anxiety; HOS – Hostility; PHOB - Phobic Anxiety; PAR - Paranoid Ideation; PSY – Psychoticism; SD – Sleep and Diet. To narrow the focus of the figure, error terms and factor loading of Psychopathology are not displayed. Here, ellipses and rectangles represent the latent and observed variables, respectively.

**Figure 3:**
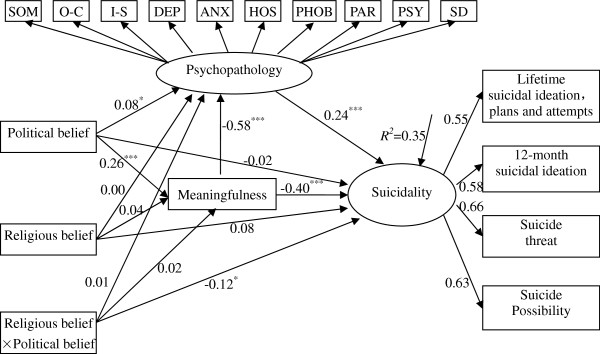
**Standardized structural coefficients for belief system, meaningfulness, psychopathology associate with suicidality among female Chinese college students.** Note: ^*^p < 0.05, ^**^p < 0.01, ^***^p < 0.001. *χ*^2^ = 614.16, *df =* 240, *χ*^2^*/df =* 2.56, *RMSEA =* 0.04, *CFI =* 0.96, *NFI =* 0.94, *IFI =* 0.96. SOM – Somatization; O-C - Obsessive-Compulsive; I-S - Interpersonal Sensitivity DEP – Depression; ANX – Anxiety; HOS – Hostility; PHOB - Phobic Anxiety; AR - Paranoid Ideation; PSY – Psychoticism;SD – Sleep and Diet. To narrow the focus of the figure, error terms and factor loading of Psychopathology are not displayed. Here, ellipses and rectangles represent the latent and observed variables, respectively.

The results of the unconstrained models are presented in Figure 2 and Figure 3. Both groups revealed that there were significant effects of political belief on meaningfulness, meaningfulness on psychopathology and suicidality, and psychopathology on suicidality. The two models indicated that political belief had an effect on suicidality through the mediation of meaningfulness, and meaningfulness to suicidality was again partially mediated by psychopathology. There were non-significant regression coefficients for both males and females: political belief to suicidality, religious belief to meaningfulness, religious belief to suicidality, political belief by religious belief to psychopathology, and political belief by religious belief to meaningfulness. Interestingly, the pathways from political belief by religious belief to suicidality differed for males and females, with the correlation reaching significance for females but not for males. Similar differences were observed in pathways from political belief to psychopathology across gender groups. However, the correlation of religious belief to psychopathology reached significance for males but not for females. Due to this pattern of results, the pathways from political belief by religious belief to suicidality,political belief to psychopathology and religious belief to psychopathology were unconstrained one at a time. The released correlation paths model allowed three pathways (political belief by religious belief to suicidality, political belief to psychopathology and religious belief to psychopathology) to remain free,but constrained all the other pathways to be equal between males and females. The fit of this model was adequate (see Table 2), *χ*^2^ = 665.74, *df* = 261, *χ*^2^/df = 2.55, RMSEA = 0.04, CFI = 0.96, NFI = 0.94, IFI = 0.96. However, the fit of the model was significantly different than that of the unconstrained model, Δ*χ*^2^ = 55.37, Δ*df* = 24*, P* = 0.000, AIC = 899.54, ECVI = 0.77 and not significantly different than that of the measurement weights invariance model (Δ*χ*^2^ = 10.13, Δ *df* = 9*, P* = 0.340) and structural weights invariance model (Δ*χ*^*2*^ = 3.80, Δ *df* = 3*, P* = 0.284). Although all four models (i.e., the unconstrained and three constrained models) yielded adequate data-model fit, we selected the unconstrained model (see Figure 2 and Figure 3) as the final model according to Δ*χ*^*2*^, AIC, and ECVI indices [48]. The results of the final models with all factor loadings can be found in Additional file 2 and 3. 

## Discussion

### Comparison of suicide prevalence with other studies

In comparison to Dai et al. [37] who gathered data among 1654 rural Chinese individuals aged 16–34 years, lifetime and one-year suicidal ideation (45.1% and 19.3% in our sample vs. 18.8% and 5.2% in that sample) and lifetime planning (6.8% in our sample vs. 5.8% in that sample) in the current sample were higher. However, our prevalence of suicidal attempts was lower than the sample by Dai et al. (1.9% vs. 2.7%). Similar patterns of discrepancy could also be found when comparing our results with Western suicide studies. For example, Nock et al. [49], using data from 17 countries, reported the lifetime prevalence of suicidal ideation, plan, and attempt as 9.2%, 3.1% and 2.7%, respectively. Our sample shows higher lifetime and past 12-month prevalence of suicidal ideation and planning, and lower lifetime prevalence of attempt. The particularly high suicidal ideation observed in the current sample could partly be explained by the study instruments used. Lifetime suicidal ideation, planning, and attempt were assessed by the first question of the SBQ: “Have you ever thought about or attempted to kill yourself?” Selecting the choice “It was just a brief passing thought” was considered a positive response for lifetime suicidal ideation. Choosing “I have had a plan at least once” was considered positive for lifetime suicidal plan, and choosing “I have attempted to kill myself” was considered positive for lifetime suicidal attempt. In the study by Dai et al. [38], lifetime suicidal ideation was recorded as positive through interviews producing any affirmative responses to questions such as “Have you ever thought about committing suicide?”. Above and beyond the difference in methodology, the demographic and psychosocial characteristics of the current sample were quite different from the rural young adult population studied by Dai et al. For example, our college students were more likely to experience disappointment in love and examination stress, whereas the rural young adults were more likely to suffer from a conflicted marriage.

### Gender differences in suicidality

The gender differences in suicidal prevalence in our sample were consistent with other Chinese studies in that females were more likely to report suicidal ideation and planning [37,50]. However, our study differed from the rural study by Dai et al. [38] in that female college students were equally likely to have had a suicidal attempt compared with their male counterparts. Gender difference patterns for suicidality as well as the higher number of suicidal ideations and plans versus lower number of suicidal attempts reported in the current sample suggest that compared with rural areas, college environments may provide some protection against suicidal ideation. It should be noted that most colleges in China have made great efforts to establish comprehensive suicide prevention systems, with a focus on the early prevention of suicidal risks. These early prevention programs, such as life education curriculum and mental health screening, may contribute to reduced suicidal attempts among Chinese young adults.

### Belief system and suicidality

The present study demonstrates that individuals’ political belief but not religious belief is protective against suicide. Compared with results in Zhang et al. [14] and Western studies [7,10,51,52], our findings provide new insights into the role of belief system on suicide prevention: in a non- or less religious population, the effect of religiosity on suicidality must be interpreted in light of the mainstream philosophical and political culture. Specifically, when one holds a political belief that is compatible with the mainstream culture, being religious can be protective against suicide. When one has no political belief, being religious can be detrimental to suicide. It is especially noteworthy that different from Taoism and Buddhism, Confucianism is closely related to and forms the basis for contemporary political beliefs among Chinese young adults, rather than being regarded as a religion [21]. It is also noted that unlike in Western religions, Chinese religious believers including Taoists, Buddhists, Christians, Muslims, and Catholics are less likely to attend religious rituals and ceremonies, are less spiritually affiliated with a religion, are more likely to believe in multiple gods, and pray for pragmatic purposes [53].

In general, Confucianism-based political beliefs advocate that one should hold a positive view toward adversities and actively commit to social responsibilities [54]. However, religion provides believers with superstitious perspectives in interpreting positive or negative life events, exerting complicated effects on believers’ spiritual well-being. Political believers are encouraged to whole-heartedly preserve life for the service of people; suicide is viewed as “dying for nothing”, and is often subject to contempt and criticism. To the contrary, belief in an afterlife, evident in most Chinese rural religious believers, might lead some individuals to commit suicide to hasten attainment of this state [13].

In a broader sense, the current study provides evidence supporting Durkheim’s social integration theory [55]. In Durkheim’s perspective, the suicide rate depends upon forces external to and constraining of individuals. If individuals fail to subordinate their own needs and desires to those of a group, the resulting lack of regulation and group ties can increase their risk for suicide. The group culture, in the case of Confucianism-based communism and socialism, renders order and meaningfulness for individuals. Integration in the form of such qualities as ties to a group and subordination to its expectations could be a powerful counteragent to suicide. At the individual level, psychological strain theory advocated by Zhang et al. [14] provides a framework for explaining the current findings, particularly the interactive effect of political and religious belief on suicidality. In the perspective of Zhang et al., suicide is an inward violence without other physical victims and could result from strains such as different values, reality vs. aspiration discrepancy, relative deprivation, and deficient coping. In Chinese society, the majority of people view going to church as deviant. Therefore, churchgoers in China may experience psychological frustration caused by religious belief that conflicts with the mainstream culture in the larger society. As found in the current study, individuals without political belief are presumably not as well adapted to the mainstream culture. According to the interpersonal psychological theory proposed by Joiner [16], they experience low belongingness and high social alienation. The internalization of religious beliefs such as believing in an afterlife and transmigration may not provide believers with adaptive coping skills, but rather instead may elicit higher degrees of value conflict within the mainstream culture. Especially for young adults whose self-identity is still developing, holding non-mainstream religious beliefs might increase internal conflict and counteract the protective effects of religiosity on suicidality. On the other hand, for political believers who are presumably well adapted to the mainstream culture, believing in religion might 1) not cause internal value conflict between different beliefs because Chinese are accustomed to believing in multiple gods and praying for pragmatic purposes [53], and 2) facilitate coping with life adversities and promote a sense of meaningfulness. By integrating pragmatism-oriented, Confucianism-based political belief with spirituality-oriented religious belief into their belief system, individuals might readily find meaningfulness in their secular life and comfort in their religious life when encountering major setbacks [56].

### Role of meaningfulness and psychopathology in suicidality

The current results suggest that meaningfulness mediates the effect of political belief on suicidality, which is further partially mediated by psychopathology. Psychopathology increased the level of suicide among those with low meaningfulness, and meaningfulness had an independent effect on suicidality after psychopathology was statistically controlled for. It should be noted that when taking meaningfulness and psychopathology into consideration, the direct impact of belief system on suicidality was reduced to non-significance (Figure 2 and Figure 3).

The current findings are generally in line with previous research. For example, one study found that low sense of meaning in life was associated with increased suicide risk even after controlling for common mental disorders [57]. Qualitative research indicated that religious belief could facilitate construction of life meaning through the act of helping others [27]. One of the essential beliefs in Confucianism-based communism and socialism is that one’s life can be fulfilled through “serving other people and the society as a whole” [24]. Political belief could motivate Chinese young adults to help others, respond positively to social issues, and accept responsibilities. By committing to social causes, the meaning of life can be achieved.

Previous findings suggest that psychopathology is the most evident risk factor for suicidality [2]. However, our findings suggest that meaningfulness can lower suicide risk even after controlling for psychopathology. In line with Philips et al. and Zhang et al. [3,4], our findings add important insights into the prevention of suicide. Previous research found that one therapeutic approach for suicide prevention is to focus on helping individuals, particularly those who are suicidal, find meaning in their lives [21]. This intervention is based on the underlying assumption that suicidal individuals question their life’s worth and have lost the desire to live without finding meaning in life. The current findings suggest that meaningfulness should be considered as an important treatment element in the effort to prevent suicide.

### Limitations and recommendations

Several limitations should be mentioned. 1) The study is cross-sectional, and the SEM does not demonstrate causation. 2) Testing differences between religious groups is essential for a thorough understanding of the effect of religiosity on suicidality. For example, Young et al. [58] found that Catholic adolescents were less likely to attempt suicide or self-harm than their Protestant peers. Unfortunately, the current sample covered only a small religious population, e.g., there were only twenty Christians (1.7%), five Muslims (0.4%), and three Catholics (0.3%). 3) Political belief and religious belief variables were narrowly defined. Future studies should develop multi-dimensional instruments to assess the internal structure of belief system as well as the strength of believers’ affiliation. 4) The effect of social support and belongingness, which presumably were associated with belief system and suicidality, were not assessed. 5) Our results could have been affected by social desirability bias because of the self-reported nature of survey. For example, suicide is undesirable in the mainstream political philosophy, and those who reported political belief are less likely to report suicidal ideation.

## Conclusions

Suicide rates and patterns vary greatly among different geographic areas and social cultures worldwide. Society and culture play significant roles in dictating how people respond to and view mental health and suicide. Culture variables, such as philosophical and ideological characteristics, may have far-reaching effects on suicidality. One’s belief system, especially the advocated ideology by mainstream culture, could influence both psychopathology and suicidality. In conclusion, in a less-religious society, political belief may serve as a means of integration as does religious affiliation in religious societies.

## End notes

^a^ Deng Xiaoping Theory is the series of political and economic ideologies first developed by Chinese leader Deng Xiaoping.

^b^ The Important Thought of Three Represents is a socio-political ideology credited to General Secretary Jiang Zemin which became a guiding ideology of the Communist Party of China at its Sixteenth Party Congress in 2002.

^c^ The Scientific Development Concept is the current official guiding socio-economic ideology of the Communist Party of China incorporating sustainable development, social welfare, a humanistic society, increased democracy, and, ultimately, the creation of a Harmonious Society.

## Competing interests

The authors declare that they have no competing interests.

## Authors' contributions

XZ designed the study and wrote the protocol. JZ and RX assisted with the survey and managed the literature searches and analyses. JZ and XY undertook the statistical analysis, wrote the first draft of the manuscript. DA and JZ edited the final manuscript. All authors read and approved the final manuscript.

## Pre-publication history

The pre-publication history for this paper can be accessed here:

http://www.biomedcentral.com/1471-2458/12/668/prepub

## Supplementary Material

Additional file 1 Mean, SD, and inter-correlations among SCL-90 subscales. Click here for file

Additional file 2 Standardized structural coefficients for belief system, meaningfulness, psychopathology associate with suicidality among male Chinese college students. Click here for file

Additional file 3 Standardized structural coefficients for belief system, meaningfulness, psychopathology associate with suicidality among female Chinese college students. Click here for file
