# Supplementary material for: Belief system, meaningfulness, and psychopathology associated with suicidality among Chinese college students: a cross-sectional survey
Source: BMC Public Health. 2012 Aug 17;12:668. doi: 10.1186/1471-2458-12-668 (PMC3491076; doi:10.1186/1471-2458-12-668)
Supplement: Additional file 1 — Mean, SD, and inter-correlations among SCL-90 subscales. [file 1471-2458-12-668-S1.doc]

### Additional file 1 –Mean, SD, and inter-correlations among SCL-90 subscales

| SCL-90  Subscales | Total sample  (n=1168,  Mean ± SD) | Male  （n=542,  Mea n± SD） | Female  （n=626,  Mean ± SD） | 1 | 2 | 3 | 4 | 5 | 6 | 7 | 8 | 9 |
| --- | --- | --- | --- | --- | --- | --- | --- | --- | --- | --- | --- | --- |
| 1 SOM | 0.25±0.34 | 0.26±0.34 | 0.24±0.35 | — |  |  |  |  |  |  |  |  |
| 2 O-C | 0.90±0.59 | 0.95±0.59 | 0.86±0.58 | 0.50^**^ | — |  |  |  |  |  |  |  |
| 3 I-S | 0.80±0.59 | 0.82±0.56 | 0.78±0.60 | 0.43^**^ | 0.70^**^ | — |  |  |  |  |  |  |
| 4 DEP | 0.56±0.52 | 0.55±0.49 | 0.57±0.54 | 0.53^**^ | 0.72^**^ | 0.72^**^ | — |  |  |  |  |  |
| 5 ANX | 0.53±0.47 | 0.54±0.46 | 0.52±0.48 | 0.63^**^ | 0.68^**^ | 0.67^**^ | 0.74^**^ | — |  |  |  |  |
| 6 HOS | 0.40±0.48 | 0.44±0.48 | 0.38±0.47 | 0.43^**^ | 0.55^**^ | 0.58^**^ | 0.56^**^ | 0.55^**^ | — |  |  |  |
| 7 PHOB | 0.39±0.45 | 0.37±0.41 | 0.42±0.49 | 0.45^**^ | 0.56^**^ | 0.63^**^ | 0.60^**^ | 0.62^**^ | 0.41^**^ | — |  |  |
| 8 PAR | 0.48±0.48 | 0.52±0.49 | 0.45±0.47 | 0.46 | 0.59^**^ | 0.66^**^ | 0.61^**^ | 0.65^**^ | 0.60^**^ | 0.46^**^ | — |  |
| 9 PSY | 0.52±0.43 | 0.52±0.43 | 0.51±0.43 | 0.51^**^ | 0.68^**^ | 0.71^**^ | 0.72^**^ | 0.68^**^ | 0.56^**^ | 0.56^**^ | 0.63^**^ | — |
| 10 SD | 0.53±0.48 | 0.54±0.49 | 0.52±0.47 | 0.55^**^ | 0.56^**^ | 0.56^**^ | 0.65^**^ | 0.63^**^ | 0.52^**^ | 0.45^**^ | 0.54^**^ | 0.62^**^ |

Note: *^*^p*<0.05, *^**^p*<0.01.

SCL-90 –Symptom Checklist-90-Revised; SOM – Somatization；O-C - Obsessive-Compulsive；I-S - Interpersonal Sensitivity；DEP – Depression；ANX – Anxiety；HOS – Hostility；PHOB - Phobic Anxiety；PAR - Paranoid Ideation；PSY – Psychoticism；SD – Sleep and Diet.
