# Supplementary material for: Belief system, meaningfulness, and psychopathology associated with suicidality among Chinese college students: a cross-sectional survey
Source: BMC Public Health. 2012 Aug 17;12:668. doi: 10.1186/1471-2458-12-668 (PMC3491076; doi:10.1186/1471-2458-12-668)
Supplement: Additional file 2 — Standardized structural coefficients for belief system, meaningfulness, psychopathology associate with suicidality among male Chinese college students. [file 1471-2458-12-668-S2.doc]

Additional file 2 - Standardized structural coefficients for belief system, meaningfulness, psychopathology associate with suicidality among male Chinese college students.

Political belief

Meaningfulness

SOM

Religious belief

Lifetime

suicidal ideation，

plans and attempts

12-month

suicidal ideation

Suicide

threat

Suicide

Possibility

0.28^***^

-0.51^***^

0.17^***^

0.10^*^

0.08

-0.08

-0.33^***^

0.55

0.61

0.49

0.69

Religious belief

×Political belief

O-C

I-S

DEP

ANX

HOS

PHOB

PAR

PSY

SD

0.02

-0.04

0.01

0.04

-0.09

*R^2^*=0.30

0.60

0.82

0.84

0.85

0.82

0.68

0.68

0.73

0.82

0.74

Note: *^*^p*<0.05， *^**^p*<0.01, *^***^p*<0.001.

*χ*^2^=614.16, *df=*240, *χ*^2^*/ df=*2.56, *RMSEA=*0.04, *CFI=*0.96, *NFI=*0.94, *IFI=*0.96.

SOM – Somatization；O-C - Obsessive-Compulsive；I-S - Interpersonal Sensitivity；

DEP – Depression；ANX – Anxiety；HOS – Hostility；PHOB - Phobic Anxiety；

PAR - Paranoid Ideation；PSY – Psychoticism；SD – Sleep and Diet.

To narrow the focus of the figure, error terms are not displayed. Here, ellipses and rectangles represent the latent and observed variables, respectively.
