# Supplementary material for: Belief system, meaningfulness, and psychopathology associated with suicidality among Chinese college students: a cross-sectional survey
Source: BMC Public Health. 2012 Aug 17;12:668. doi: 10.1186/1471-2458-12-668 (PMC3491076; doi:10.1186/1471-2458-12-668)
Supplement: Additional file 3 — Standardized structural coefficients for belief system, meaningfulness, psychopathology associate with suicidality among female Chinese college students. [file 1471-2458-12-668-S3.doc]

Political belief

Meaningfulness

Religious belief

Lifetime

suicidal ideation，

plans and attempts

12-month

suicidal ideation

Suicide

threat

Suicide

Possibility

0.24^***^

-0.58^***^

0.26^***^

0.00

0.08

-0.12^*^

-0.40^***^

0.55

0.63

0.58

0.66

Religious belief

×Political belief

0.08^*^

-0.02

0.04

0.02

0.01

*R^2^*=0.35

SOM

O-C

I-S

DEP

ANX

HOS

PHOB

PAR

PSY

SD

0.66

0.82

0.86

0.88

0.86

0.65

0.71

0.74

0.83

0.72

Additional file 3 - Standardized structural coefficients for belief system, meaningfulness, psychopathology associate with suicidality among female Chinese college students.

Note: *^*^p*<0.05， *^**^p*<0.01, *^***^p*<0.001.

*χ*^2^=614.16, *df=*240, *χ*^2^*/ df=*2.56, *RMSEA=*0.04, *CFI=*0.96, *NFI=*0.94, *IFI=*0.96.

SOM – Somatization；O-C - Obsessive-Compulsive；I-S - Interpersonal Sensitivity；

DEP – Depression；ANX – Anxiety；HOS – Hostility；PHOB - Phobic Anxiety；

PAR - Paranoid Ideation；PSY – Psychoticism；SD – Sleep and Diet.

To narrow the focus of the figure, error terms are not displayed. Here, ellipses and rectangles represent the latent and observed variables, respectively.
